# Serologic Presentation of Lamotrigine-Induced Lupus

**DOI:** 10.1155/2022/8756308

**Published:** 2022-09-29

**Authors:** Hannah Dempsey, Gabriella Aitcheson, Gretchen Goble, Riley Snook

**Affiliations:** Indiana University School of Medicine, Indianapolis, IN, USA

## Abstract

This paper discusses the presentation of a rare drug side effect, a case of drug-induced lupus presenting with weight loss, weakness, hepatitis, and pancreatitis. A 24-year-old male with a history of major depressive disorder and childhood seizures presented to the ER with symptoms of abdominal pain, significant weight loss, and weakness. Initial workup revealed acute pancreatitis, elevated liver function enzymes (LFTs), and abnormal anti-double-stranded DNA antibody (anti-dsDNA) 1 : 640. He showed no classical clinical signs of lupus including rash, arthritis, or photosensitivity. He had multiple hospitalizations in the previous 6 months for excessive weight loss, malnutrition, weakness, and altered mental status. He had been taking lamotrigine for seizure prevention and mood stabilization while on a selective serotonin reuptake inhibitor (SSRI) and had a decline in health since the lamotrigine dose was increased. Antihistone antibodies were positive suggesting a drug-induced lupus syndrome. We hope to bring awareness to the possible rare complication of lamotrigine-induced lupus.

## 1. Introduction

Lamotrigine is primarily an antiseizure medication that inhibits voltage-dependent sodium channels and decreases presynaptic glutamate and aspartate release. It has also been used as a mood stabilizer for people with bipolar disorder. Its side effect profile includes common reactions such as nausea, vomiting, anorexia, headaches, and insomnia as well as more rare, serious reactions such as hemophagocytic lymphohistiocytosis (HLH), Steven–Johnson syndrome (SJS), toxic epidermal necrolysis (TEN), and aseptic meningitis. A review of the literature shows a handful of case reports of drug-induced lupus connected with lamotrigine and more recently in the last few years. We present a case of drug-induced lupus (DIL) that presents with symptomatic sequelae of the disease such as weight loss, weakness, altered mental status, elevated LFTs, and pancreatitis.

## 2. Case

A cachectic appearing 24-year-old male presented as a transfer from an outside hospital with symptoms of acute onset abdominal pain, nausea, and nonbloody, nonbilious vomiting. He denies arthralgias, rash, or oral ulcers. The patient had no history of alcohol or illicit drug use. He had had no work exposure, recent travel, or sick contacts. He had been treated with lamotrigine in the past for childhood seizure disorder but had not been taking it for years. He was prescribed sertraline and lamotrigine 100 mg in Feb 2020 as a treatment for his major depression and suicidal ideation. The dose was recently increased to 100 mg BID in Feb 2021 to better control symptoms. The patient noted a gradual decline in health since Dec 2020. He lost 30 pounds, despite normal appetite. He had numerous hospitalizations in the prior 6 months for symptoms of altered mental status and muscle pain and weakness to the point of needing a walker for unsteady gait. Workup included upper and lower endoscopy and bone marrow biopsy due to changing cytopenia, but the results were all reportedly normal.

Upon presentation to our institution, he was afebrile and normotensive with a heart rate of 107. He had no oxygen requirements. Of note, he had a body mass index (BMI) of 15, and autoimmune labs at an outside hospital were positive for antinuclear antibody (ANA), anti-dsDNA, anti-Smith, and anti-smooth muscle antibodies. A physical exam showed a chronically ill young man with epigastric abdominal pain and general weakness, but no focal neurological deficits. He had no obvious rash or oral ulcers. Blood work showed elevated lipase up to 2682 and triglycerides of 693 at an outside hospital as well as elevated liver enzymes with alkaline phosphatase (ALP) of 822, aspartate aminotransferase (AST) 661, and alanine transaminase (ALT) 121, total bilirubin 9.6, albumin 2.0, international normalized ratio (INR) 1.26. At their lowest, white blood cell count 2.6 (91% neutrophils), platelets 16 k, Hemoglobin 6.6, reticulocyte count 0.9%, sodium 129, potassium 2.9, creatinine 0.26. New autoimmune labs were ordered per Rheumatology consult to evaluate for systemic lupus erythematosus (SLE). ANA titer 1 : 640, complement component (*C*3) low 26, with normal complement component (*C*4), anti-Smith ab titer of 1 : 180, positive chromatin antibody. Anti-PL 7 and anti-Jo-1 were positive on the cytoplasmic ANA pattern website, but negative on the ANA panel. A right upper quadrant ultrasound was negative for gallstones, bile duct dilation, or portal vein thrombosis as a cause for elevated LFTs. A magnetic resonance cholangiopancreatography (MRCP) showed diffusely edematous pancreas with ascites and liver changes consistent with hepatitis and pancreatitis. A liver biopsy showed steatohepatitis with focal pericellular fibrosis and no signs of cirrhosis. Computed tomography (CT) of the chest showed pericarditis and loculated bilateral pleural effusions. An echocardiogram performed for occasional chest pain showed left ventricular hypokinesis with an ejection fraction of 48%. Troponins were normal.

Initial medical management included fluids, bowel rest, and nutritional support. His abdominal pain and pancreatitis resolved. Blood work suggested an autoimmune process with SLE in the differential. Since he did not have the classic clinical presentation of SLE, we were concerned about a drug-induced etiology for this autoimmune type of picture. We reviewed the patient's recent medications in the last 6 months. He had been on chronic lamotrigine for his depression and mood disorder and the dose was increased in the last few months. A positive antihistone antibody suggested a drug-induced process. The lamotrigine was discontinued, and the patient was treated with blood transfusions and 3 days of intravenous methylprednisolone. He had marked clinical improvement as well as normalization of LFT, complete blood count (CBC), reticulocyte count, and ANA panel. His appetite improved and he reported more energy. He was discharged on a steroid taper and started on hydroxychloroquine at a later rheumatology check-up.

## 3. Discussion

This case highlighted the difficulties surrounding a lupus diagnosis with multiple different diagnostic tests, but it was unique because of the lack of characteristic lupus clinical signs. The presentation of lupus, in this case, was almost solely a lab diagnosis, leading to even more confusion as to the cause of the symptoms he did portray.

The patient's age was consistent with the optimal age range for SLE and the clinical presentation technically fits the SLE classification criteria with 5/11 clinical criteria (serositis, neurologic symptoms, hemolytic anemia, leukopenia, and thrombocytopenia) and 5/6 immunologic criteria (positive ANA, dsDNA, anti-Smith, low complement, and positive direct coombs test) [1]. Ultimately, primary SLE was deemed less likely by the rheumatology consult in this case due to the lack of classic cutaneous rash, oral ulcers, or arthralgias. However, the strong laboratory signs of SLE and the presentation consistent with autoimmune pancreatitis/hepatitis, as well as general weakness made us look further into other similar etiologies, including drug-induced lupus. A look at his medication list sparked an interest in lamotrigine as the possible culprit.

The timing of his symptoms in relation to starting lamotrigine and the presence of antihistone antibodies, suggests drug-induced lupus (DIL). Antihistone antibodies are positive in 95% of DIL cases and only 50% of SLE [2]. An autoimmune or drug-induced hepatitis could also explain the patient's abnormal LFTs. A score of 5 on the adverse drug reaction probability scale suggests a moderately high possibility that this patient may have a drug-induced liver injury [3].

A literature search for other cases of lamotrigine-induced lupus was completed. Four such cases were found. The first case was a 22-year-old female with symptoms of rash, photosensitivity, and oral ulcers with labs showing positive ANA, but negative antihistone and dsDNA antibodies [4]. The second case was an 18-year-old female with symptoms of rash, oral ulcers, and arthralgias as well as labs with positive ANA and again negative for antihistone and dsDNA antibodies [5]. The third case involved a 57-year-old female with symptoms of rash, photosensitivity, arthralgias and myalgias, and Raynaud's. She had positive ANA and negative rheumatoid factor [6]. The last case was of a 39-year-old male only presenting with symptoms of small joint arthralgias, but with strongly positive ANA titers and once again, negative dsDNA [7]. All of these cases had varying dosages ranging from 50 mg/day [4] to 600 mg/day [7] and all had their symptoms decrease or resolve once lamotrigine was removed and they were given steroids.

Our patient did not show any of the classic signs that these other rare cases presented such as rash, oral ulcers, or arthralgias. However, his labs responded quickly to the cessation of lamotrigine and added prednisone. His physical symptoms of weakness and confusion slowly improved as well with additional nutrition and lamotrigine cessation. His BMI increased from 15 to 18.9 in the span of 2 months. Although drug-induced lupus is rare, we need to consider this in a patient taking lamotrigine who comes in with clinical or laboratory signs of lupus.

## Data Availability

The data supporting the current study are given in the article.
